# Alcohol use during pregnancy in Rakai, Uganda

**DOI:** 10.1371/journal.pone.0256434

**Published:** 2021-08-26

**Authors:** Adriane Wynn, Dorean Nabukalu, Tom Lutalo, Maria Wawer, Larry W. Chang, Susan M. Kiene, David M. Serwadda, Nelson Sewankambo, Fred Nalugoda, Godfrey Kigozi, Jennifer A. Wagman

**Affiliations:** 1 Division of Infectious Diseases & Global Public Health, Department of Medicine, University of California, San Diego, La Jolla, CA, United States of America; 2 Rakai Health Sciences Program/Uganda Virus Research Institute, Kalisizo, Uganda; 3 Department of Epidemiology, Johns Hopkins Bloomberg School of Public Health, Baltimore, MD, United States of America; 4 Department of Medicine, John Hopkins School of Medicine, Baltimore, MD, United States of America; 5 Division of Epidemiology and Biostatistics, School of Public Health, San Diego State University, San Diego, CA, United States of America; 6 Makerere University School of Public Health, Kampala, Uganda; 7 Makerere University School of Medicine, Kampala, Uganda; 8 Department of Community Health Sciences, UCLA Fielding School of Public Health, Los Angeles, CA, United States of America; SUNY Downstate Health Sciences University, UNITED STATES

## Abstract

**Introduction:**

Antenatal alcohol use is linked to adverse maternal and neonatal outcomes. Uganda has one of the highest rates of alcohol use in sub-Saharan Africa, but the prevalence of antenatal alcohol use has not been reported in the Rakai region.

**Methods:**

We used cross-sectional data from pregnant women in the Rakai Community Cohort Study between March 2017 and September 2018. Using bivariate and multivariable analyses, we assessed associations between self-reported antenatal alcohol use and sociodemographic characteristics, intimate partner violence (IPV), and HIV status.

**Results:**

Among 960 pregnant women, the median age was 26 years, 35% experienced IPV in the past 12 months, 13% were living with HIV, and 33% reported alcohol use during their current pregnancy. After adjusting for marital status, education, smoking, and HIV status; Catholic religion (AOR: 3.54; 95% CI: 1.89–6.64; compared to other), bar/restaurant work (AOR: 2.40; 95% CI: 1.17–4.92; compared to agriculture), >one sex partner in past year (AOR: 1.92; 95% CI: 1.17–3.16), a partner that drank before sex in past year (AOR: 2.01; 95% CI: 1.48–2.74), and past year IPV (AOR: 1.55; 95% CI: 1.14–2.11) were associated with antenatal alcohol use.

**Conclusion:**

We found that alcohol use during pregnancy was common and associated with religion, occupation, higher numbers of past year sex partners, having a partner who drank before sex in the past 12 months, and IPV experience. More research is needed to understand the quantity, frequency, and timing of antenatal alcohol use; and potential impacts on neonates; and to identify services that are acceptable and effective among pregnant women.

## Introduction

Alcohol use is an important cause of morbidity and mortality worldwide [1], and sub-Saharan Africa (SSA) bears the largest alcohol-attributable burden of disease and injury [2]. Alcohol use during pregnancy is common in many countries with one systematic review and meta-analysis estimating the worldwide prevalence to be 9.8% (8.9–11.1%) [3]. Antenatal exposure to alcohol is a global public health concern associated with adverse health consequences for women and neonates, including stillbirth, low birthweight, preterm birth, and fetal alcohol spectrum disorders (FASD) [4,5].

Uganda has one of the highest rates of alcohol use in SSA [2]. The World Health Organization (WHO) estimated that, in 2016, alcohol use per capita was 9.5 liters of pure alcohol (16.1 among men and 3.0 among women) [2]. Although 63.7% of the population (aged 15 years and older) reported that they abstained from alcohol use in the past year, among those who drank, 56.9% reported heavy episodic drinking (68.8% among men and 32.6% among women), and 7.1% met criteria for alcohol use disorders (12.4% among men and 1.9% among women) [2]. Studies of antenatal alcohol exposure in Uganda are limited. A 2010 study found that among a sample of pregnant women seeking antenatal care at the national referral hospital in Kampala (Uganda’s capital city), 25% reported alcohol use after learning they were pregnant [6]. Another study that combined self-reported alcohol use and a blood biomarker found that 20% of pregnant women living with HIV in southwestern Uganda met the criteria for hazardous alcohol use [7].

Previous research in Rakai, Uganda, the setting for this research, found a high prevalence of alcohol use, including 39% among all women surveyed [8] and 42% among young women [9]. However, prevalence and correlates of alcohol use among pregnant women have not been assessed in Rakai. Understanding the patterns and drivers of alcohol use during pregnancy is important for developing interventions to reduce antenatal alcohol consumption, FASD, and interrelated problems such as HIV infection and intimate partner violence (IPV). This paper utilizes cross-sectional data collected in 2017–2018 to estimate the prevalence and correlates of antenatal alcohol use among pregnant women in Rakai, Uganda.

## Methods

### Study population

This cross-sectional study was conducted among pregnant women in the mostly rural Rakai region of southwestern Uganda. The data for this study were derived from the Rakai Community Cohort Study (RCCS) conducted between March 2017 and September 2018. RCCS is an open, prospective HIV surveillance cohort which takes place in Rakai, Uganda and has been described in detail previously [10]. Since 1994, RCCS has enrolled approximately 15,000 consenting participants aged 15–49 years every 12–20 months. Prior to each RCCS surveillance survey, a household census is conducted to identify all community members eligible for enrollment. Thereafter, all present community residents who are within the eligible age range and have provided informed consent are surveyed as part of RCCS. At each survey visit, participants are interviewed regarding sociodemographic and behavioral information and voluntary venous blood is obtained for HIV testing. Participants who provide written informed consent are interviewed in central community locations (referred to as “hubs”). Those not captured at the hubs are approached at their household or place of work to request their participation. Up to two return visits are made in an attempt to enroll eligible participants. Interviews are administered by same sex interviewers who use a structured questionnaire with questions on sexual behaviors, sexual partners, health status and service utilization, and reproductive health. Free HIV post-test education and services are offered to participants who choose to give blood, including results to all consenting individuals and couples.

### Eligibility criteria

Data were eligible for inclusion in our analytic sample if the participant was a woman who self-reported as pregnant through an affirmative response to the survey question, “Are you pregnant now?” and who responded to the question about alcohol use during pregnancy. Although pregnancy status was confirmed by human chorionic gonadotropin (HCG) urine test for women uncertain about their pregnancy status, alcohol use during pregnancy was only assessed among those who self-reported as pregnant. Data were excluded from: (1) Women who self-reported as pregnant, but did not answer the alcohol use during pregnancy question (n = 1); and (2) Women who did not self-report as pregnant, but were later found to be pregnant via HCG urine test (n = 130). Among women who self-reported as pregnant, 213 who were not visibly pregnant were also given an HCG test, and 21 tested negative. Our sample includes all women who self-reported as pregnant and thus includes the 21 who later tested negative. We compared sociodemographic characteristics and alcohol responses between those who tested negative and those tested positive for pregnancy and found no differences.

### Measures

We assessed socio-demographic information, including age, religion, education level, occupation, and marital status. Rakai communities are also categorized as agrarian, trading or fishing. Agrarian communities are often a great distance from main roads and the most commonly reported primary occupation is agriculture and/or production and maintenance of crops and farmland. Trading communities are often close to main roads, have high mobility, and the most commonly reported primary occupation is buying and selling of goods and/or services. Fishing communities are residential areas with landing sites on Lake Victoria where the primary occupation relates to harvesting or processing of fishery resources [11]. Education level was measured using a dichotomous variable where those with zero to the seven years of primary school were defined as “full primary school or less” and those with more than seven years were defined as “secondary or higher.” A household social economic status (SES) index was compiled based on dwelling structure (e.g. grass thatch roofs and mud floors were categorized as low SES) using methods developed for use in Rakai and previously described [12]. We also measured past year cigarette smoking (yes/no). HIV testing was performed using a validated three rapid test algorithm. Women living with HIV responded to the questions: “Have you ever been to a clinic to receive care for HIV?” and “Are you currently taking this medication?” (i.e., ARVs). Sexual behaviors included number of past year sex partners (none, 1, ≥2). Participants reported on whether alcohol was used by her partner(s) prior to sex in the past 12 months. IPV victimization was assessed by reports of emotional, physical, or sexual abuse by an intimate partner within the past 12 months using an adapted version of the Conflict Tactics Scale [13]. Emotional IPV was measured by asking if a partner verbally abused or shouted at the participant. Physical IPV was measured by asking if a partner perpetrated any of the following behaviors against the participant: pushing, pulling, grabbing, kicking, slapping, punching, burning, strangling, and/or attacking with a weapon. Sexual IPV was measured by asking if a partner perpetrated any of the following sexually abusive behaviors against the participant: physically forcing her to have sex or forcing her to perform sexual activities when she did not want to.

The main alcohol use variable examined in this analysis was alcohol use during pregnancy, defined as ‘Have you been drinking alcohol during this pregnancy?’ (yes/no). Participants were also asked about their past year alcohol use, defined as ‘Have you drunk any alcohol in the past year, for instance, beer, wine, waragi or other spirits, or home-made beer?’ (yes/no). Participants who reported affirmatively to past year alcohol use were subsequently asked the following questions:

*To assess alcohol-related aggression and violence*: 1) In the past year, when you drank alcohol, did you get angry, get violent or get into a fight? (yes/no for each item).

*To assess loss of control related to drinking*: “How often during the last year have you felt you should cut down on your drinking or stop altogether?” (Never, occasionally, sometimes, often; which was recoded to Never and occasionally or more)

*To assess alcohol’s impact on daily responsibilities and activities*: “In the past year, have you ever taken alcohol while you were at work?” (yes/no)

*To assess harmful alcohol use, we used three items from the Alcohol Use Disorders Identification Test (AUDIT) [14]*:

- **Guilt after drinking**: In the past year, when you drank alcohol, have you ever have felt ashamed of something that you did while drinking? (yes/no)
- **Blackouts:** In the past year, when you drank alcohol, did you ever forget some of the things you did or that happened while you were drinking? (yes/no)
- **Others concerned about drinking:** Has a relative or friend, doctor or other health worker been concerned about your drinking or suggested that you cut down? (yes/no)

*To assess alcohol dependence*:

- Using criteria from the International Classification of Diseases (WHO, 1993): “In the past year, when you drank alcohol, did you ever experience an unsteady gait? Fall over? Have difficulty speaking? Have shaking hands the next morning?” (yes/no for each item)
- Using one item from the AUDIT scale [14]: “How often during the last year have you failed to do something that you wanted or needed to do because of your drinking?” (Never, occasionally, sometimes, often; which was recoded to Never and occasionally or more).

### Analysis

We estimated the prevalence of alcohol use during pregnancy, including a confidence interval based on a binomial distribution. Correlates of alcohol use during pregnancy were assessed using Fisher’s exact or Chi-square tests for categorical variables and Wilcoxon rank-sum or Student’s t-test for continuous variables. We also fit logistic regression models to calculate the unadjusted odds ratios (ORs) and 95% confidence intervals (CIs) to estimate associations between alcohol use and key sociodemographic, sexual behavior variables (e.g. number of partners), and HIV status. Next, we fit an adjusted logistic regression model, which included covariates that were significantly associated with alcohol use during pregnancy at the P<0.05 level in bivariate analyses and findings from prior research in Uganda demonstrating a relationship between alcohol use, HIV and IPV [15]. We ran collinearity diagnostics using a Variance Inflation Factor (VIF) of ≥5 or a tolerance of ≤0.1 to signal the presence of multi-collinearity. In our models, multicollinearity was ruled out because tolerance measures were above 0.85 and all VIFs did not exceed two.

We also stratified the alcohol-related consequence variables by alcohol use during pregnancy and experience of past year IPV and conducted bivariate comparisons using Fisher’s exact or Chi-square tests for categorical variables and Wilcoxon rank-sum or Student’s t-test for continuous variables.

### Ethics

All study instruments and protocols were reviewed and approved by the Western IRB, the Uganda Virus Research Institute’s Research and Ethics Committee (UVRI-REC) and the Uganda National Council of Science and Technology (UNCST). The plan for analysis and publication of these data was reviewed and approved by the University of California (UC) San Diego Human Research Protections Program (HRPP), the UC Los Angeles HRPP, and UVRI-REC. All RCCS participants provided written consent to take part in the study and all were compensated 10,000 Ugandan shillings (approximately $3 USD) for their time and transport refund.

## Results

In our sample of 960 women who reported they were pregnant at the time of interview, 33% (95% CI: 30–36%) reported that they used alcohol during their current pregnancy. Table 1 displays sociodemographic characteristics, HIV status, HIV care and ART uptake, number of sex partners, whether partners drank before sex, and IPV experiences stratified by alcohol use during pregnancy. The median age was 26 years, 81% were currently married, 63% had completed full primary school or less, and 59% were Roman Catholic. The most commonly reported occupation among women was in agriculture (41%) followed by trader or shopkeeper (24%) and housework (in own home) (16%). The largest proportion of participants (55%) were drawn from agrarian communities; 24% were from trading centers, and 21% were from fishing villages. Only 2% of women reported that they currently smoked.

**Table 1:** Characteristics by alcohol use during pregnancy among participants in RCCS between March 2017 and September 2018.

	Total Sample	Have you been drinking during this pregnancy?		
	(N = 960)	Yes (n = 319) (33%)	No (n = 641) (67%)	
	No. (col %)	No (row %)	No (row %)	P-value
**Age (years), Median [range]**	26 [15–46]	26 [16–46]	26 [15–42]	0.409
15–24 yrs	425 (44)	137 (32)	288 (68)	
25–34 yrs	399 (42)	133 (33)	266 (67)	
35+ yrs	136 (14)	49 (36)	87 (64)	
**Marital status**				**0.021**
Currently married	786 (81)	255 (32)	531 (68)	
Previously married	101 (11)	45 (45)	56 (55)	
Never	73 (8)	19 (26)	54 (74)	
**Education**				**0.02**
Primary or less	602 (63)	217 (36)	385 (64)	
Secondary or higher	358 (37)	102 (28)	256 (72)	
**Religion**				**<0.001**
Other	80 (8)	15 (19)	6 (81)	
Catholic	568 (59)	228 (40)	340 (60)	
Protestant	166 (17)	47 (28)	119 (72)	
Muslim	146 (15)	29 (20)	117 (80)	
**Occupation**				**0.004**
Agriculture	391 (41)	125 (32)	266 (68)	
Housework or Housekeeper	149 (16)	59 (40)	90 (60)	
Clerical/Teacher	62 (6)	12 (19)	50 (81)	
Trader/Shop keeper	229 (24)	77 (34)	152 (66)	
Bar or Restaurant	41 (4)	22 (54)	19 (46)	
Other	88 (9)	24 (27)	64 (73)	
**Household SES**				0.225
High	633 (66)	210 (33)	423 (67)	
Middle	195 (20)	58 (30)	137 (70)	
Low	131 (14)	51 (39)	80 (61)	
**Community type**				0.067
Agrarian	532 (55)	177 (33)	355 (67)	
Trading	230 (24)	65 (28)	165 (72)	
Fishing	198 (21)	77 (39)	121 (61)	
**Currently smoke**				**0.002**
Yes	18 (2)	12 (67)	6 (33)	
No	942 (98)	307 (33)	635 (67)	
**HIV Status**				**0.026**
Positive	127 (13)	53 (42)	74 (58)	
Negative	822 (87)	261 (32)	561 (68)	
**Ever had HIV care** (HIV positive)				0.718
Yes	119 (94)	49 (41)	70 (59)	
No	8 (6)	4 (50)	4 (50)	
**Currently on ART** (HIV positive)				0.236
Yes	115 (91)	46 (40)	69 (60)	
No	12 (9)	7 (58)	5 (42)	
**Number of sex partners past year**				**<0.001**
1	864 (90)	266 (31)	598 (69)	
2+	95 (10)	52 (55)	43 (45)	
**Partner drank before sex past year**				
Yes	375 (39)	174 (46)	201(54)	**<0.001**
No	584 (61)	145 (25)	439 (75)	
**Intimate Partner Violence (IPV) past year**				**<0.001**
No IPV	662 (65)	174 (28)	448 (72)	
Any IPV	337 (35)	145 (43)	192 (57)	
Emotional IPV	308 (32)	136 (44)	172 (56)	
Physical IPV	217 (23)	96 (44)	121 (55)	
Sexual IPV	69 (7)	30 (43)	39 (57)	

Notes: Ever had HIV care and currently on ART are among those living with HIV. IPV p-value is between none and any IPV. Pregnant at time of abuse and partner drank at time of abuse are among those who experienced emotional, physical or sexual abuse.

The HIV prevalence was 13%. Among women living with HIV, 94% ever had HIV care and 91% reported that they were on ART at the time of interview. Regarding sex partners, 10% reported having two or more sex partners in the past 12 months, and 39% reported that partner(s) drank alcohol before sex in the past 12 months. In the past year, 35% of women reported that they experienced some form of IPV, including emotional (32%), physical (23%), and sexual (7%).

In the unadjusted models, drinking during pregnancy was associated (at the p<0.05 level) with being previously married, having a lower level of education, Catholic religion, being employed at a bar or restaurant, past year smoking, having two or more sex partners in the past year, having a partner that drank before sex in the past year, living with HIV, and experiencing IPV in the past year (Table 2). In the adjusted model, Catholic religion (AOR: 3.54; 95% CI: 1.89–6.64), having a job in housework (AOR: 1.69; 95% CI: 1.09–2.61), having a job at a bar or restaurant (AOR: 2.40; 95% CI: 1.17–4.92), having two or more sex partners in the past year (AOR: 1.92; 95% CI: 1.17–3.16), having a partner that drank before sex in the past year (AOR: 2.01; 95% CI: 1.48–2.74), and past year IPV (AOR: 1.55; 95% CI: 1.14–2.11) were associated with alcohol use during pregnancy. Marital status, education level, past year smoking, and HIV status were no longer associated with alcohol use during pregnancy after adjusting for covariates.

**Table 2:** Unadjusted and adjusted logistic regression models of correlates of alcohol use during pregnancy among participants in RCCS between March 2017 and September 2018.

	Unadjusted Estimate			Adjusted Estimate		
	Odds Ratio	95% CI	p-value	Odds Ratio	95% CI	p-value
**Marital status**						
Never married	Ref					
Previously married	2.28	(1.19–4.39)	**0.01**	1.47	(0.72–3.02)	0.29
Currently married	1.36	(0.79–2.35)	0.26	1.16	(0.65–2.08)	0.62
**Education**						
≤Primary	Ref					
≥Secondary	0.71	(0.53–0.94)	**0.02**	0.85	(0.61–1.18)	0.33
**Religion**						
Other	Ref					
Catholic	2.91	(1.62–5.22)	**<0.001**	3.54	(1.89–6.64)	**<0.001**
Protestant	1.71	(0.89–3.30)	0.12	1.94	(0.96–2.80)	0.06
Muslim	1.07	(0.54–2.15)	0.2	1.34	(0.63–2.80)	0.77
**Occupation**						
Agriculture	Ref					
Housework	1.4	(0.94–2.06)	0.1	1.69	(1.09–2.61)	**0.02**
Clerical/Teacher	0.51	(0.26–0.99)	**0.05**	0.76	(0.37–1.59)	0.47
Trader/Shop	1.08	(0.76–1.53)	0.67	1.34	(0.91–1.97)	0.13
Bar or Restaurant	2.46	(1.29–4.72)	**0.007**	2.4	(1.17–4.92)	**0.02**
Other	0.8	(0.48–1.34)	0.39	0.84	(0.48–1.48)	0.55
**Currently smoke**						
No	Ref					
Yes	4.14	(1.54–11.13)	**0.005**	2.82	(0.98–8.10)	**0.05**
**HIV Infected**						
Uninfected	Ref					
Infected	1.54	(1.05–2.26)	**0.03**	1.01	(0.66–1.56)	0.96
**Number of sex partners past year**						
1	Ref					
2+	2.77	(1.80–4.25)	**<0.001**	1.92	(1.17–3.16)	**0.01**
**Partner drank before sex**						
No	Ref					
Yes	2.62	(1.99–3.46)	**< .001**	2.01	(1.48–2.74)	**<0.001**
**Intimate Partner Violence (IPV)**						
No IPV	Ref					
Any IPV	1.94	(1.47–2.57)	**< .001**	1.55	(1.14–2.11)	**0.005**

Among pregnant women who reported drinking in the past year (n = 386, 40%), 96 (25%) reported that they did not drink during their current pregnancy and 280 (73%) reported experiencing no alcohol-related consequence (Table 3). The most common consequences of alcohol use were related to alcohol dependence and impact on daily responsibilities, including feeling they should cut down on drinking (34%), failing to do something they wanted or needed to do (13%), drinking at work (13%), and having an unsteady gait (12%). Few significant differences emerged in terms of alcohol-related consequences between past year drinkers who did and did not consume alcohol during their pregnancy. One exception is that women who reported drinking in the past year but *not* during their pregnancy were more likely to report an unsteady gait in the past year (19% compared to 10%). We also compared the reported consequences of past year alcohol use by IPV experience (among women who drank in the past year). Women who experienced IPV had significantly higher proportions of alcohol consequences compared to those who didn’t experience IPV, with the exception of the variable “did you ever fall over,” where only three people said yes. Among women who drank in the past year and experienced IPV, the three most commonly reported alcohol-related consequences were feeling they should cut down on their drinking (42%), failing to do something they wanted or needed (21%), and getting angry (20%).

**Table 3:** Consequences of alcohol use among RCCS participants between March 2017 and September 2018 who reported to be pregnant and used alcohol in the past year, by alcohol use during pregnancy and IPV.

	Total	Used alcohol during pregnancy			Experienced IPV, past year		
	N = 386	Yes (n = 290)	No (n = 96)		Yes (n = 172)	No (n = 214)	
		n (%)	n (%)	p-value	n (%)	n (%)	p-value
**No consequences**	280 (73)	210 (72)	70 (73)	0.518	97 (56)	183 (86)	<0.001
**Alcohol-Related Aggression & Violence**							
*In the past year when you drank alcohol*, *did you ever*:							
Get angry	37 (10)	30 (10)	7 (7)	0.378	34 (20)	3 (1)	<0.001
Get violent/in a fight	30 (8)	27 (9)	3 (3)	0.052	27 (16)	3 (1)	<0.001
**Harmful Alcohol Use**							
*In the past year when you drank alcohol*, *did you ever*:							
Feel ashamed of something done	25 (6)	18 (6)	7 (7)	0.708	12 (12)	4 (2)	<0.001
Forget things you did/happened	18 (5)	15 (5)	3 (3)	0.41	15 (9)	3 (1)	0.001
**Alcohol Dependence**							
*In the past year when you drank alcohol*, *did you ever*:							
Have an unsteady gait	46 (12)	28 (10)	18 (19)	0.017	33 (19)	13 (6)	<0.001
Fall over	3 (1)	3 (1)	0 (0)	0.317	3 (2)	0 (0)	0.088
Have difficulty speaking	12 (3)	8 (3)	4 (3)	0.491	12 (7)	0 (0)	<0.001
Have shaking hands the next morning	12 (3)	8 (3)	4 (4)	0.491	10 (6)	2 (1)	0.007
Fail to something wanted/needed	51 (13)	40 (14)	11 (11)	0.566	35 (21)	16 (7)	<0.001
**Loss of Control Related to Drinking**							
In the past year, did you feel you should cut down on your drinking or stop?	133 (34)	104 (36)	29 (30)	0.312	72 (42)	61 (29)	0.007
**Alcohol’s Impact on Daily Responsibilities and Activities**							
In the past year have you taken alcohol while at work?	49 (13)	42 (14)	7 (7)	0.067	28 (16)	21 (10)	0.066

Note: *p-values were derived from Fisher’s Exact tests and reflect the relationship between alcohol consequences and those who did and did not report IPV (emotional, physical, and/or sexual).

## Discussion

### Our findings

We found that the prevalence of self-reported drinking during pregnancy in Rakai, Uganda, was over three times higher than the global average of alcohol use among pregnant women [3], higher than previous studies conducted among pregnant women in other regions of Uganda [6,7], but lower than the prevalence of alcohol use among non-pregnant women in Rakai, Uganda [8]. Correlates of alcohol use during pregnancy were Catholic religion, occupation in a restaurant or bar, higher numbers of past year sex partners, having a partner who drank before sex in the past 12 months, and experiencing IPV. Among women who reported drinking in the past year, consequences of alcohol use (e.g. feeling you should cut down drinking, failing to something you needed because of drinking, and getting angry when drinking) were more common among women who also experienced IPV during the past year, compared to women who drank in the past year but did *not* experience IPV.

Our findings bolster previous results demonstrating the co-occurrence of and interrelationship between alcohol use and syndemics such as IPV, depression, and HIV infection among non-pregnant women in SSA [15–17]. A recent study found that 47% of non-pregnant women attending a hospital outpatient clinic and eligible for HIV testing in rural Uganda reported having one or more of the following conditions: depression, emotional or physical IPV, and/or alcohol use [15]. This study also found evidence of synergistic effects of multiple conditions to increase HIV risk. Women experiencing two or more conditions (compared to none) reported more high-risk sex acts (AOR: 2.18; 95% CI: 1.64–2.91) and had greater odds of testing positive for HIV or an STI (AOR: 5.87; 95% CI: 1.99–17.35). Previous research among women in Rakai found that alcohol use before sex was associated with HIV incidence (one partner drank: adjIRR 1.40, 95% CI 1.02–1.92; both partners drank: adjIRR 1.81, 95% CI 1.34–2.45), physical IPV (at least one partner drank: AOR 1.68; 95% CI: 1.41–2.01) [9], and sexual coercion (at least one partner drank: AOR 1.85, 95% CI 1.48–2.31) [18]. A study in South Africa found that mothers with depression, who experienced IPV, and/or who were HIV infected were more likely to drink alcohol [19]. The accumulation of health problems among women in SSA demonstrates a need to understand how multiple conditions interact to increase risks for adverse outcomes.

### Next steps

Our finding of high alcohol use during pregnancy in Rakai, Uganda suggests the need for additional research on maternal alcohol use, neonatal health outcomes, and interventions to address these global public health problems. First, we recommend future research on maternal alcohol use utilize a standardized, validated psychometric tool to measure alcohol consumption quantity and frequency. According to the U.S. Centers for Disease Control and Prevention, there is no known safe amount of alcohol use during pregnancy and no safe time during pregnancy to drink [20]. However, quantity, frequency, and timing impact risks for adverse health outcomes, including HIV transmission and FASD. A recent systematic review and meta-analysis found that disease and mortality outcomes associated with alcohol use were often accelerated in a dose-response relationship [21]. This study also found that heavy drinking levels and alcohol use disorders were associated with viral load increases, which were partly mediated by treatment non-adherence [21]. Another study found that, compared to no reported drinking during pregnancy, the odds of FASD increased with the number of trimesters that a woman used alcohol [22]. This study also found that binge drinking was associated with increased risk for FASD. Thus, research that includes drinking patterns will be important for developing appropriate prevention strategies.

The burden of FASD in SSA may be large, but it is not well measured. FASD is an umbrella term describing the range of effects that can occur when an individual is antenatally exposed to alcohol, with the most serious being fetal alcohol syndrome [23]. FASD is associated with serious and long-term effects on children [5,23], which can result in significant economic impact [24]. There are currently no data on FASD in Uganda [25]. One modeling study estimated that the prevalence of FASD in Uganda was 16.2 (95% CI: 10–24.3) per 1000 population [25]. Given our finding that a large proportion of pregnant women reported drinking during pregnancy, there is an urgent need to generate awareness and increase training in FASD diagnosis in order to increase surveillance and link children to care.

Maternal alcohol use and corresponding adverse outcomes are preventable. Thus, implementing screening, brief interventions, and referrals for alcohol use and IPV could result in important benefits for women and children in Uganda [17,19]. While some interventions have shown promise for reducing harmful alcohol use among pregnant women [26,27], including Case Management for women at high risk for having a child with FASD; more research is needed demonstrating intervention effectiveness in Uganda [28,29]. Additionally, there is some evidence showing alcohol reduction policies and programs are cost-effective [30,31]; however, among pregnant women, the research is limited. In low resource settings, it may be possible to prioritize interventions for women with high risk for using alcohol during pregnancy. For example, we found that pregnant women who work in bars or restaurants were at increased risk. Further, leveraging antenatal care infrastructure, integrating multiple services to address the syndemics of alcohol use, IPV, and HIV; and including community-based support and task-shifting may increase the value of programs that have the potential to promote healthier pregnancies in SSA.

This study had several limitations. First, our data were cross-sectional and we were unable to assess causality in the relationships between alcohol use during pregnancy and correlates. Next, alcohol use, number of sex partners, and IPV were self-reported and participants might have underestimated or forgotten their true behaviors, potentially leading to reduced accuracy of findings. However, it is encouraging to note that our alcohol use findings are similar to previous studies, some of which used biomarkers [7]. Third, our analysis is among women who self-reported as pregnant and our sample included some women who were not pregnant and excluded women who were unknowingly pregnant. Thus, our findings represent the behavior of women who believed they were pregnant; however, longitudinal studies that include neonatal health outcomes should include participants with confirmed pregnancies. Fourth, the alcohol consequence and alcohol use by sex partners questions reflect alcohol use over the past year and we were unable to determine whether they took place during pregnancy or whether behavior changed after the pregnancy. Finally, although our alcohol use measures were based on items from the internationally validated AUDIT, the questions were not taken verbatim and the full tool was not included in RCCS. Thus, we were unable to fully capture the quantity or frequency of alcohol consumed and related severity or intensity of alcohol use. We recommend future studies utilize standardized, validated psychometric tools for alcohol use such as the AUDIT, AUDIT-C, TWEAK (Tolerance, Worried, Eye-opener, Amnesia, Cut Down) and the Substance Use Risk Profile-Pregnancy (SURP-P) as well as an alcohol biomarker to improve the validity of the measurement.

## Conclusion

This study fills an important gap in the literature by estimating the prevalence and correlates of alcohol use during pregnancy using a recent cohort of participants in the RCCS. We found that alcohol use during pregnancy was common and was associated with religion, occupation, higher numbers of past year sex partners, having a partner who drank before sex in the past 12 months, and IPV experience. More research is needed to understand the quantity, frequency, and timing of antenatal alcohol use; and potential impacts on neonates; and to identify programs and services that are acceptable and effective among pregnant women.

## References

[1] GBD 2016 Alcohol Collaborators. Alcohol use and burden for 195 countries and territories, 1990–2016: a systematic analysis for the Global Burden of Disease Study 2016. Lancet. 2018;392(10152):1015–35. doi: 10.1016/S0140-6736(18)31310-2 30146330 PMC6148333

[2] World Health Organization. Global status report on alcohol and health 2018. Licence: CC BY-NC-SA 30. 2018;Accessed from: http://apps.who.int/iris/bitstream/handle/10665/274603/9789241565639-eng.pdf?ua=1.

[3] Popova S, Lange S, Probst C, Gmel G, Rehm J. Estimation of national, regional, and global prevalence of alcohol use during pregnancy and fetal alcohol syndrome: a systematic review and meta-analysis. The Lancet Global Health. 2017;5(3):e290–e9. doi: 10.1016/S2214-109X(17)30021-9 28089487

[4] Patra J, Bakker R, Irving H, Jaddoe VW, Malini S, Rehm J. Dose-response relationship between alcohol consumption before and during pregnancy and the risks of low birthweight, preterm birth and small for gestational age (SGA)-a systematic review and meta-analyses. BJOG: an international journal of obstetrics and gynaecology. 2011;118(12):1411–21. 21729235 10.1111/j.1471-0528.2011.03050.x PMC3394156

[5] Lange S, Rovet J, Rehm J, Popova S. Neurodevelopmental profile of Fetal Alcohol Spectrum Disorder: A systematic review. BMC psychology. 2017;5(1):22. doi: 10.1186/s40359-017-0191-2 28645298 PMC5481937

[6] Namagembe I, Jackson LW, Zullo MD, Frank SH, Byamugisha JK, Sethi AK. Consumption of alcoholic beverages among pregnant urban Ugandan women. Maternal and child health journal. 2010;14(4):492–500. doi: 10.1007/s10995-009-0500-3 19629663 PMC2906221

[7] Raggio GA, Psaros C, Fatch R, Goodman G, Matthews LT, Magidson JF, et al. High Rates of Biomarker-Confirmed Alcohol Use Among Pregnant Women Living With HIV in South Africa and Uganda. J Acquir Immune Defic Syndr. 2019;82(5):443–51. doi: 10.1097/QAI.0000000000002156 31567551 PMC6857734

[8] Wagman JA, Nabukalu D, Miller AP, Wawer MJ, Ssekubugu R, Nakowooya H, et al. Prevalence and correlates of men’s and women’s alcohol use in agrarian, trading and fishing communities in Rakai, Uganda. PLoS One. 2020;15(10):e0240796. doi: 10.1371/journal.pone.0240796 33125397 PMC7598464

[9] Zablotska IB, Gray RH, Koenig MA, Serwadda D, Nalugoda F, Kigozi G, et al. Alcohol use, intimate partner violence, sexual coercion and HIV among women aged 15–24 in Rakai, Uganda. AIDS Behav. 2009;13(2):225–33. doi: 10.1007/s10461-007-9333-5 18064556

[10] Wawer MJ, Sewankambo NK, Serwadda D, Quinn TC, Paxton LA, Kiwanuka N, et al. Control of sexually transmitted diseases for AIDS prevention in Uganda: a randomised community trial. Rakai Project Study Group. Lancet. 1999;353(9152):525–35. doi: 10.1016/s0140-6736(98)06439-3 10028980

[11] Chang LW. Heterogeneity of the HIV epidemic: an observational epidemiologic. 2016;3(8):e388–96. 10.1016/S2352-3018(16)30034-0 PMC4973864 27470029

[12] Makumbi FE, Nakigozi G, Sekasanvu J, Lukabwe I, Kagaayi J, Lutalo T, et al. Incidence of orphanhood before and after implementation of a HIV care programme in Rakai, Uganda: Alpha Network HIV Supplement. Tropical medicine & international health: TM & IH. 2012;17(8):e94–102. 22716203 10.1111/j.1365-3156.2012.03031.x PMC4169214

[13] Straus M. Measuring Intrafamily conflict and violence: the conflict tactics (CT) scales. J Marriage Fam. 1979;41(1):75–88.

[14] Babor T, Higgins-Biddle J, Saunders J, Monteiro M. AUDIT: The Alcohol Use Disorders Identification Test: Guidelines for use in primary care (2nd ed.). Geneva, Switzerland: World Health Organization. 2001.

[15] Kiene SM, Lule H, Sileo KM, Silmi KP, Wanyenze RK. Depression, alcohol use, and intimate partner violence among outpatients in rural Uganda: vulnerabilities for HIV, STIs and high risk sexual behavior. BMC infectious diseases. 2017;17(1):88. doi: 10.1186/s12879-016-2162-2 28103834 PMC5248514

[16] Russell BS, Eaton LA, Petersen-Williams P. Intersecting epidemics among pregnant women: alcohol use, interpersonal violence, and HIV infection in South Africa. Current HIV/AIDS reports. 2013;10(1):103–10. doi: 10.1007/s11904-012-0145-5 23233038 PMC3572769

[17] Culley CL, Ramsey TD, Mugyenyi G, Kiwanuka GN, Ngonzi J, Macleod S, et al. Alcohol exposure among pregnant women in sub-saharan Africa: a systematic review. Journal of population therapeutics and clinical pharmacology = Journal de la therapeutique des populations et de la pharamcologie clinique. 2013;20(3):e321–33. 24163173

[18] Zablotska IB, Gray RH, Serwadda D, Nalugoda F, Kigozi G, Sewankambo N, et al. Alcohol use before sex and HIV acquisition: a longitudinal study in Rakai, Uganda. Aids. 2006;20(8):1191–6. doi: 10.1097/01.aids.0000226960.25589.72 16691071

[19] Davis EC, Rotheram-Borus MJ, Weichle TW, Rezai R, Tomlinson M. Patterns of Alcohol Abuse, Depression, and Intimate Partner Violence Among Township Mothers in South Africa Over 5 Years. AIDS Behav. 2017;21(Suppl 2):174–82. doi: 10.1007/s10461-017-1927-y 29027039 PMC5839633

[20] Centers for Disease Control and Prevention. Fetal Alcohol Spectrum Disorders (FASDs): Alcohol Use in Pregnancy 2020 [Available from: https://www.cdc.gov/ncbddd/fasd/alcohol-use.html.

[21] Rehm J, Gmel GE Sr., Gmel G, Hasan OSM, Imtiaz S, Popova S, et al. The relationship between different dimensions of alcohol use and the burden of disease-an update. Addiction (Abingdon, England). 2017;112(6):968–1001. doi: 10.1111/add.13757 28220587 PMC5434904

[22] May PA, Blankenship J, Marais AS, Gossage JP, Kalberg WO, Joubert B, et al. Maternal alcohol consumption producing fetal alcohol spectrum disorders (FASD): quantity, frequency, and timing of drinking. Drug and alcohol dependence. 2013;133(2):502–12. doi: 10.1016/j.drugalcdep.2013.07.013 23932841 PMC3829200

[23] Riley EP, Infante MA, Warren KR. Fetal alcohol spectrum disorders: an overview. Neuropsychology review. 2011;21(2):73–80. doi: 10.1007/s11065-011-9166-x 21499711 PMC3779274

[24] Greenmyer JR, Klug MG, Kambeitz C, Popova S, Burd L. A Multicountry Updated Assessment of the Economic Impact of Fetal Alcohol Spectrum Disorder: Costs for Children and Adults. Journal of addiction medicine. 2018;12(6):466–73. doi: 10.1097/ADM.0000000000000438 30383615

[25] Lange S, Probst C, Gmel G, Rehm J, Burd L, Popova S. Global Prevalence of Fetal Alcohol Spectrum Disorder Among Children and Youth: A Systematic Review and Meta-analysis. JAMA pediatrics. 2017;171(10):948–56. doi: 10.1001/jamapediatrics.2017.1919 28828483 PMC5710622

[26] Wechsberg WM, Bonner CP, Zule WA, van der Horst C, Ndirangu J, Browne FA, et al. Addressing the nexus of risk: Biobehavioral outcomes from a cluster randomized trial of the Women’s Health CoOp Plus in Pretoria, South Africa. Drug and alcohol dependence. 2019;195:16–26. doi: 10.1016/j.drugalcdep.2018.10.036 30562676 PMC6415667

[27] Hutton HE, Chander G, Green PP, Hutsell CA, Weingarten K, Peterson KL. A novel integration effort to reduce the risk for alcohol-exposed pregnancy among women attending urban STD clinics. Public health reports (Washington, DC: 1974). 2014;129 Suppl 1:56–62. doi: 10.1177/00333549141291S109 24385650 PMC3862990

[28] Ospina M, Moga C, Dennett L, Harstall C. Prevention of Fetal Alcohol Spectrum Disorder FASD. A Systematic Review of the Effectiveness of Prevention Approaches for Fetal Alcohol Spectrum Disorder. Weinheim, Germany.: Wiley-VCH Verlag GmbH & Co.; 2015. p. 99–335.

[29] Sileo KM, Miller AP, Wagman JA, Kiene SM. Psychosocial interventions for reducing alcohol consumption in sub-Saharan African settings: a systematic review and meta-analysis. Addiction (Abingdon, England). 2021;116(3):457–73. doi: 10.1111/add.15227 33463834 PMC8543382

[30] Anderson P, Chisholm D, Fuhr DC. Effectiveness and cost-effectiveness of policies and programmes to reduce the harm caused by alcohol. Lancet. 2009;373(9682):2234–46. doi: 10.1016/S0140-6736(09)60744-3 19560605

[31] Chisholm D, Moro D, Bertram M, Pretorius C, Gmel G, Shield K, et al. Are the "Best Buys" for Alcohol Control Still Valid? An Update on the Comparative Cost-Effectiveness of Alcohol Control Strategies at the Global Level. Journal of studies on alcohol and drugs. 2018;79(4):514–22. 30079865

